# Vγ9Vδ2 T Cells Activation Through Phosphoantigens Can Be Impaired by a RHOB Rerouting in Lung Cancer

**DOI:** 10.3389/fimmu.2020.01396

**Published:** 2020-07-07

**Authors:** Chloé Laplagne, Sarah Meddour, Sarah Figarol, Marie Michelas, Olivier Calvayrac, Gilles Favre, Camille Laurent, Jean-Jacques Fournié, Stéphanie Cabantous, Mary Poupot

**Affiliations:** ^1^Centre de Recherches en Cancérologie de Toulouse, Inserm UMR1037, Toulouse, France; ^2^Université Toulouse III Paul-Sabatier, Toulouse, France; ^3^ERL 5294 CNRS, Toulouse, France; ^4^IUCT-O, Toulouse, France

**Keywords:** RHOB, Vγ9Vδ2 T cells, phosphoantigen, endosomes, split-GFP, TCR activation

## Abstract

Vγ9Vδ2 T cells are known to be efficient anti-tumor effectors activated through phosphoantigens (PAg) that are naturally expressed by tumor cells or induced by amino bisphosphonates treatment. This PAg-activation which is TCR and butyrophilin BTN3A dependent can be modulated by NKG2D ligands, immune checkpoint ligands, adhesion molecules, and costimulatory molecules. This could explain the immune-resistance observed in certain clinical trials based on Vγ9Vδ2 T cells therapies. In NSCLC, encouraging responses were obtained with zoledronate administrations for 50% of patients. According to the *in vivo* results, we showed that the *in vitro* Vγ9Vδ2 T cell reactivity depends on the NSCLC cell line considered. If the PAg-pretreated KRAS mutated A549 is highly recognized and killed by Vγ9Vδ2 T cells, the EGFR mutated PC9 remains resistant to these killers despite a pre-treatment either with zoledronate or with exogenous BrHPP. The immune resistance of PC9 was shown not to be due to immune checkpoint ligands able to counterbalance NKG2D ligands or adhesion molecules such as ICAM-1 highly expressed by PC9. RHOB has been shown to be involved in the Vγ9Vδ2 TCR signaling against these NSCLC cell lines, in this study we therefore focused on its intracellular behavior. In comparison to a uniform distribution of RHOB in endosomes and at the plasma membrane in A549, the presence of large endosomal clusters of RHOB was visualized by a split-GFP system, suggesting that RHOB rerouting in the PC9 tumor cell could impair the reactivity of the immune response.

## Introduction

Gamma delta (γδ) T lymphocytes expressing the T cell receptor (TCR) Vγ9Vδ2 are a prominent γδ T cell subset in human peripheral blood representing 1–3% of blood mononuclear cells. Upon activation with non-peptide phosphoantigens (PAgs), these Vγ9Vδ2 T cells proliferate, produce chemokines and cytokines, and mediate cell cytotoxicity against a large spectrum of tumor cells (1). These molecules are metabolites from the methyl erythritol phosphate pathway in microbial pathogens (2) and from the eukaryotic mevalonate pathway in tumor cells which are thus spontaneously recognized and killed by Vγ9Vδ2 T cells (3, 4). In humans, treatment with aminobisphosphonates such as zoledronate can exacerbate Vγ9Vδ2 T cell reactivity through the upregulation of the endogenous biosynthesis of PAgs in mammalian cells (5). This PAgs activation was clearly shown to be TCR-dependent. However, as Vγ9Vδ2 T cells express different activator and inhibitor receptors such as immune checkpoint inhibitors and natural killer (NK) receptors, their reactivity can also be exacerbated or curbed by ligands expressed by target cells (6, 7). Thus, even overproduction of endogenous PAgs might reflect the metabolic biases of cancer cells, and presumably occurs in most if not all types of tumors, some of which are resistant to Vγ9Vδ2 T killing. Accordingly, tumor-infiltrating γδ T cells have been detected in several solid and hematopoietic malignancies but are not always correlated with a good prognosis (8, 9). However, these cells remain very attractive candidates for cancer immunotherapies regarding a tumor regression associated with their significant amplification in the blood for some clinical trials based on PAg treatment or adoptive transfers of Vγ9Vδ2 T cells (10–12). In patients with non-small cell lung cancer (NSCLC), administration of zoledronate was correlated with an increase of Vγ9Vδ2 T cells in blood and a higher overall survival (13). A phase I clinical study showed the safety and potential anti-tumor effect of reinfused *ex-vivo* expanded Vγ9Vδ2 T cells in patients with advanced NSCLC refractory to or intolerant to current conventional treatment (14). These partial responses and the inevitable relapse with classical treatments make NSCLC incurable pathologies for which many mechanisms of acquired resistance have been elucidated, but the recurrent immune-resistance remains obscure. RHOB is a known tumor suppressor in lung cancer, and its downregulation, frequently observed in aggressive tumors (15), is associated with decreased overall survival (16). More recently, RHOB has also been shown to confers resistance to EGFR-tyrosine kinase inhibitors in NSCLC (17), suggesting different roles of this GTPase depending on the oncogenic and/or therapeutic context. Interestingly, RHOB was recently shown to mediate endogenous PAg recognition by the Vγ9Vδ2 TCR (18). RHOB interaction with endogenous PAg in the target cell could induce a modification of the conformation of the membrane butyrophilin BTN3A1 which then activates the Vγ9Vδ2 TCR (19). Here, we investigated the role of RHOB in the response to PAg-mediated γδ T cell activation in two NSCLC cell lines with the most represented oncogenic mutations KRAS and EGFR. After showing that A549 was well-recognized and killed by Vγ9Vδ2 T cells compared to PC9, we found different patterns of surface molecule expression for these two NSCLC cell lines. However, the resistance of PC9 to Vγ9Vδ2 T cell killing could be due to a rerouting of RHOB in late/degradation compartments that may prevent its function with BTN3A1 at the plasma membrane in PC9 cells.

## Materials and Methods

### Reagents and Antibodies

Antibodies for flow cytometry analysis: BV310 anti-CD3, FITC anti-TCRVγ9Vδ2, PE or PeCy5 anti-CD107a, PeCy7 anti-IFNγ, PE anti-TIM3, PE anti-Galectin9, PeCy7 anti-PD1, APC anti-PDL1, PeCy5 anti-CD80, PE anti-CD80, PeCy5 anti-HLAABC, AF647 anti-CD31, PeCy7 anti-CD38, FITC anti-CD226, FITC anti-CD112, FITC anti-CD155, PE anti-LFA1, and isotype controls (BD Biosciences, Pont de Claix, France); BV421 anti-CD69 and isotype control (Miltenyi Biotech, Paris, France); PE anti-HLAE (eBiosciences); PE anti-ULPB2,5,6 (R&D Systems, Minneapolis, USA); APC anti-MICA/B (Biolegend, St-Quentin-en-Yvelines, France); PE anti-ICAM1 and PE anti-ICAM3 (Immunotech, Marseille, France); PE anti-LFA3 (Beckman Coulter, Fullerton, CA, USA).

Blocking antibodies: anti-BTN3A1 1 h at 10 μg/mL (103.2 clone, kindly gifted by ImCheck Therapeutics, Marseille, France), anti-γδTCR 1 h at 0.5 mg/mL (B1 clone, Biolegend), anti-ICAM1 (W-CAM-1 clone, Thermo fisher, Villebon sur Yvette, France) and anti-CD31 1 h at 10 μg/mL (HEC7 clone, Thermo fisher, Villebon sur Yvette, France). The exoenzyme C3 transferase was used as RHO inhibitor I overnight at 2 μg/mL (Cytoskeleton, Inc. Denver, USA).

### Flow Cytometry Analysis

Cells were labeled with 5 μg/ml antibodies or isotype controls for 20 min at 4°C and analyzed on an LSRII cytometer (BD Biosciences, Pont de Claix, France). Data were analyzed using BD FACSDiva software, FlowJo software or FlowLogic software.

### Vγ9Vδ2 T Cell Cultures

Primary Vγ9Vδ2 T cell cultures were generated from peripheral blood mononuclear cells (PBMCs) isolated from blood of healthy donors (Etablissement Français du Sang, Toulouse, France). Briefly, PBMC were stimulated with BrHPP (3 μM) and rhIL-2 (300 IU/ml) in complete RPMI 1640 culture medium (Invitrogen, Cergy Pontoise, France) supplemented with 10% fetal calf serum (Hy1, Thermo Scientific, USA), 100 g/ml streptomycin, 100 IU/ml penicillin and 1 mM sodium-pyruvate (Cambrex Biosciences, Rockland, ME, USA) for 14 days. Purity of the Vγ9Vδ2 T cells was >95% as determined by flow cytometry using an anti-TCRVγ9Vδ2 mAb.

### Lung Cancer Cell Lines

The human NSCLC cell lines A549, H1299, H827, and PC9 were previously obtained from the American Type Culture Collection (Manassas, VA, USA) and cultured in RPMI 1640 medium containing 10% fetal bovine serum (FBS) and were maintained at 37°C in a humidified chamber containing 5% CO_2_.

For the RHOB KO A549 and PC9, the TALEN sequences targeting RHOB were designed by CELLECTIS (Paris, France), and inserted into two plasmids comprising CMV and T7 promoters. Triple transfection of the two TALEN-encoding plasmids with a Puromycin selection cassette upstream RHOB gene was performed using the JetPrime® transfection agent (Polyplus transfection) according to the manufacturer's recommendations. Puromycin selection was performed for 48 h after transfection and the pool of surviving clones was subcloned by limit dilution in 96-well plates. For each subclone, RHOB DNA levels and RHOB protein expression were analyzed by PCR and Western Blot.

### Cytotoxicity Assay

Lung cancer cell lines were treated at 70% of confluence with BrHPP (1 μM, 4 h, Innate Pharma, Marseille, France) or Zoledronic acid monohydrate (Zometa, 5 μM, overnight, Sigma Aldrich, Saint Louis, USA). After washing, treated cancer cells were co-cultured in 96-wells plates with overnight IL-2-deprived Vγ9Vδ2 T cells (E:T ratio 1:1) in complete medium with anti-CD107a mAb or IgG1 control (5 μg/ml). Brefeldin A (10 μg/ml, Sigma Aldrich, St Quentin Fallavier, France) was added at 2 h of co-culture. After 4 h of co-culture cells were washed, stained and analyzed by flow cytometry. When mentioned, contact between γδ T cells and target cells was prevented thanks to a Transwell® system (Corning). For intracellular IFNγ expression, cells were fixed with PBS 2% paraformaldehyde and permeabilized with PBS containing 5% FCS and 1% saponin (Sigma-Aldrich) prior to staining for 30 min with the specified mAb for flow cytometry analysis.

### Trogocytosis Analysis

Lung cancer cells were stained with the lipophilic green-emitting dye PKH67 (Sigma-Aldrich, Saint Louis, USA) according to the manufacturer's instructions. Then, PKH67-positive cells were co-cultured for 4 h in complete culture medium with PKH67-negative Vγ9Vδ2 T cells in 96-well U-bottom culture plates at a cell ratio of 1:1. After gentle centrifugation (110 g for 1 min) and co-cultures for 3 min or 4 h at 37°C, cells were washed with 0.5 mM PBS/EDTA. Trogocytosis was measured as the acquisition of PKH67 fluorescence, which was characterized via the increase of the mean fluorescence intensity (mfi) of PKH67 by a flow cytometry.

### Monitoring RHOB Activity With a Split-GFP Reporter System

To evaluate the effect of PAg, A549, and PC9 cell lines were engineered to express the tripartite split-GFP biosensor system previously developed to monitor RHOB activity in single cells (20). To generate the split-GFP reporter cell line, sequential transductions were performed with lentiviruses encoding for GFP1-9, the detector fragment of the split-GFP system and the chimeric construct that coexpress both RHOB fused to the strand 10 of trisfGFP (GFP10-RHOB) and the RHO-binding domain of Rhotekin (RBD) fused to strand 11 of trisfGFP (RBD-GFP11) in A549-rtTA or PC9-rtTA cells. After recovery, cells were induced with 0.25 μg/mL doxycycline for 24 h and sorted by FACS based on GFP fluorescence. To improve the GFP fluorescence signal upon split-GFP complementation, a GFP nanobody (21) was expressed from a lentiviral expression vector on optimized cell lines.

For both fluorescence quantifications of RHOB activity and analysis of RHOB localization, reporter cells were grown on μ-Slide 8-well ibiTreat chambered coverslips (Ibidi, Biovalley). Cells were seeded at a density of 15,000 cells/well for PC9 and 35,000 cells/well for A549 for 24 h. Split-GFP reporter expression was induced for 24 h with 0.25 μg/ml Doxycycline in RPMI culture medium supplemented with 2% serum (PC9) and 10% serum (A549), and subsequently treated with PAg for 16 h with Zoledronic acid monohydrate or 4 h of BrHPP. To stop the experiment, cells were fixed with 4% PFA, PBS then stained with a cytoplasmic cell mask, HCS CellMaskTM Blue Stain (Thermo Fisher Scientific) according to the supplier's instructions. Quantitative image acquisition was performed using an Operetta high-content imaging system (Perkin Elmer) with a 20× objective lens in the 488/525 nm (GFP) and 360/405 nm (cell mask) channels. Analysis was performed with Harmony® software on an average of 1500 cells/well. The number of objects and the sum of cell area was determined from the cell mask staining. The percentage of GFP cells was calculated as: percentage GFP cells = (number of GFP-positive cells/number of all objects) ×100, where GFP-positive cells are defined by cells following this criteria: mean of fluorescence intensity (MFI) of the object>mean of MFI in the control wells without doxycycline. GFP intensity sum/cell area was defined as the (GFP intensity sum of GFP+ cells)/(HCS intensity sum of GFP+ cells).

For confocal analysis, cells were fixed with 3.7% PFA and permeabilized with 0.1% Triton X-100 in PBS buffer. Blocking was performed with 8% BSA, PBS for 30 min before adding primary antibodies. Anti-GFP10 polyclonal antibody (20) was used at 1:1,000 dilution for 1 h, followed by secondary antibody Alexa fluor 594 conjugate anti rabbit IgG (Life technologies) for 40 min. For labeling endosomes, the following primary antibodies were used: Rab7 [(D95F2) XP 9367, Cell signaling] 1:50, LAMP1 (H5G11) sc-18821, Santa Cruz Biotechnology 1:50. After overnight incubation, secondary antibodies were added Alexa fluor 594 conjugate anti rabbit IgG (Life technologies) and Alexa fluor 647 conjugate anti mouse IgG (Life technologies). Microscopy images were acquired using LSM 780 or LSM 880 (Zeiss, Oberkochen, Germany) confocal laser scanning microscopes using a 488 Argon laser with a 490–553 nm emission filter (GFP) Alexa 594 and DAPI labeling were acquired with Argon laser (543 nm) and 405 UV diode lasers, respectively, using 20x and 63× /1.4 oil immersion objectives. Image analysis was performed with ImageJ® software.

### Statistical Analysis

Data are expressed as means ± SEM. For comparison of two series of normally distributed variables, we used paired and one-tailed Student's *t*-tests with α = 0.05 for statistical significance. Statistical analysis were performed with Prism software.

## Results

### Differential Activation of Vγ9Vδ2 T Cells by Different PAg-Treated NSCLC Cell Lines

**Figure 1:**
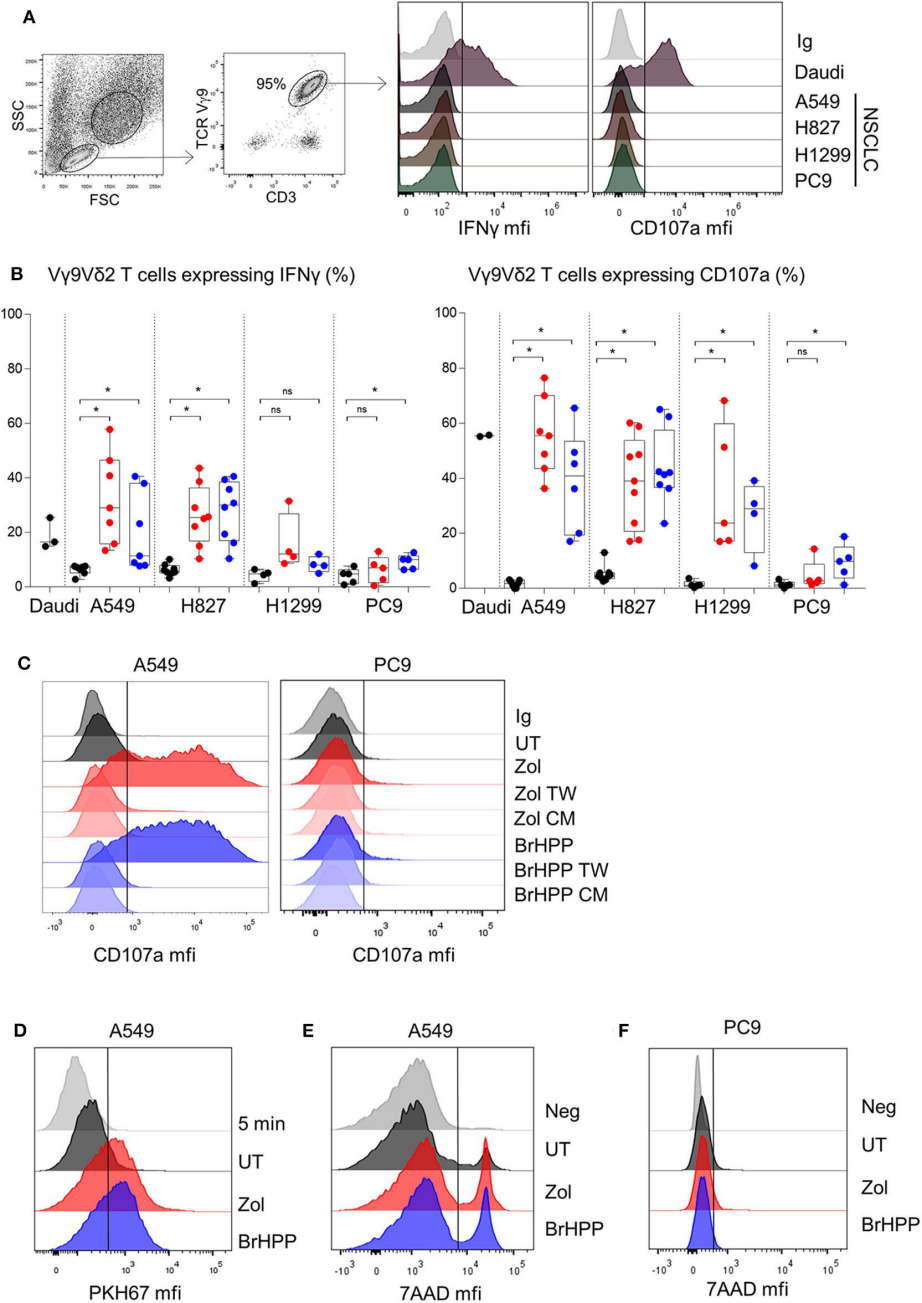
PAg-treated NSCLC cell lines activate Vγ9Vδ2 T cells in a contact dependent manner. Flow cytometry analysis of the IFNγ and CD107a expression by Vγ9Vδ2 T cells in co-culture for 4 h with four different NSCLC cell lines without pretreatment **(A)** or with zoledronate (red dots) or BrHPP (blue dots) pretreatment [**(B)**, *n* > 4 independent experiments], with Transwell system or tumor cell lines conditioned medium in case of A549 and PC9 as target **(C)**. Flow cytometry analysis of the trogocytosis of the PKH67^+^ PAg-treated A549 cell line by the Vγ9Vδ2 T cells after 5 min or 4 h of co-culture, i.e., PKH67 expression by the Vγ9Vδ2 T cells **(D)**. Measure by flow cytometry of the 7-AAD positive PAg-treated-A549 **(E)** or -PC9 **(F)** after co-culture with Vγ9Vδ2 T cells for 4 h. *indicates *p* < 0.05, Student's paired *t*-test; ns: no significant.

To analyze the role of RHOB in Vγ9Vδ2 T cell reactivity against NSCLC, we first screened the basal reactivity of Vγ9Vδ2 T cells against four cell lines with different mutation statuses: A549 (KRAS mutant), H1299 (HRAS mutant), PC9 and H827 (EGFR mutant). The expression of CD107a and IFNγ by Vγ9Vδ2 T cells after co-cultures with the different tumor cell lines was measured by flow cytometry. Compared to the Daudi control target cells, which induced CD107a and IFNγ expression by Vγ9Vδ2 T cells, no basal reactivity was detected with the four NSCLC cell lines (Figures 1A,B black dots). However, incubation of these cell lines with zoledronate (red dots) or with an exogenous synthetic PAg, BrHPP (blue dots), induced a high increase of the percentage of IFNγ and CD107a positive Vγ9Vδ2 T cells in co-culture with A549, H827, and H1299 but not with PC9 (Figure 1B). We then checked that this reactivity was due to an immunological synapse following a contact between Vγ9Vδ2 T cells and NSCLC target cells and not to PAg excreted in the co-culture medium. Vγ9Vδ2 T cells were thus incubated for 4 h with A549 or PC9, previously treated with PAg, and separated by a Transwell (TW) membrane or with their conditioned medium. As shown in Figure 1C, no CD107a expression was detected in Vγ9Vδ2 T cells in the TW condition or in the conditioned medium condition (CM) (Figure 1C for a representative experiment and Supplementary Figure 1 for pooled experiments). Trogocytosis was also evaluated to confirm the contact dependent reactivity of Vγ9Vδ2 T cells against A549. Trogocytosis is the transfer of membrane patches following the establishment of an immunological synapse between a T lymphocyte and a target cell. The membrane transfer was measured by the increase of green (PKH67) fluorescence expressed by the Vγ9Vδ2 T following contact with the A549 cell line previously stained with the PKH67 fluorescent probe stably inserted into the plasma membrane. Vγ9Vδ2 T cells expressed PKH67 fluorescence after 4 h of contact with PAg-treated PKH67^+^A549 compared to 5 min of contact (Figure 1D for a representative experiment and Supplementary Figure 2 for pooled experiments). CD107a and IFNγ expression by Vγ9Vδ2 T cells, and their trogocytosis in reaction to A549 were correlated with the death of A549 (Figure 1E and Supplementary Figure 3A). On the contrary, PC9, even treated by phosphoantigens was relatively resistant to Vγ9Vδ2 T cell killing (Figure 1F and Supplementary Figure 3B).

Vγ9Vδ2 T cells can thus be highly activated to kill A549 but not PC9, when previously treated with phosphoantigens.

### Expression of Different Patterns of Surface Ligands and Adhesion Molecules on A549 and PC9

**Figure 2:**
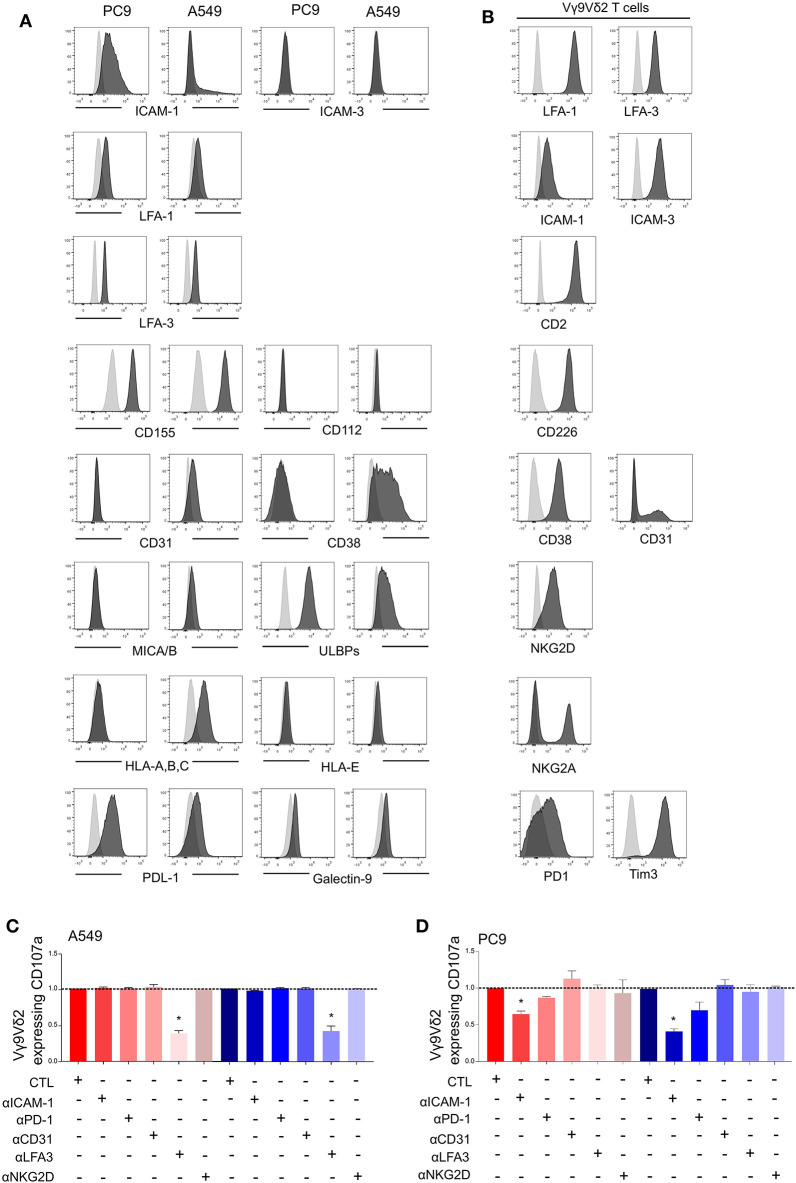
Vγ9Vδ2 T cells activation by PAg-treated-A549 or -PC9 is dependent on different adhesion molecules. Flow cytometry analysis of the expression of surface markers (black) by PC9 and A549 cell lines **(A)** or by the Vγ9Vδ2 T cells **(B)** compared to the respective isotypic control (gray). CD107a expression by the Vγ9Vδ2 T cells after 4 h of co-culture with zoledronate-treated A549 [**(C)**, red] or BrHPP-treated A549 [**(C)**, blue] or with zoledronate-treated PC9 [**(D)**, red] or BrHPP-treated PC9 [**(D)**, blue] in the presence of different blocking antibodies normalized to the control condition (CTL) without blocking antibody. *indicates *p* < 0.05.

The immunological synapse between Vγ9Vδ2 T cells and tumor cells involves different surface molecules such as activator and inhibitor ligands/receptors, and adhesion molecules. Expression of these molecules at the surface of A549, PC9, and the Vγ9Vδ2 T cells was performed by flow cytometry analysis. With regard to the adhesion molecule pattern, LFA-1, LFA-3, and CD155 were expressed at the same level by A549 and PC9 whilst ICAM-3, CD112, CD31, and the costimulatory ligands CD80/CD86 were not expressed (Figure 2A and Supplementary Figure 4A). However, PC9 expressed high levels of the adhesion molecule ICAM-1 compared to A549, and A549 expressed high level of CD38 compared to PC9. The corresponding receptors were checked at the surface of Vγ9Vδ2 T cells which thus expressed LFA-1, ICAM-1, ICAM-3, CD2, LFA-3, CD226, CD38, and CD31 (Figure 2B). Considering the activator and inhibitor ligands, A549 expressed less ULBPs and PDL1 than PC9 but more HLA-A,B,C whereas MICA/B and galectin-9 were not expressed neither by A549 nor by PC9 (Figure 2A), Vγ9Vδ2 T cells expressed the corresponding receptors NKG2A, NKG2D, PD1, and Tim-3 (Figure 2B). The level of expression of all these ligands/receptors on A549 and PC9 were not statistically affected by PAg treatment (Supplementary Figure 4B). We then investigated which surface molecules were involved in Vγ9Vδ2 T cell activation using blocking antibodies. We showed that blocking the LFA-3/CD2 axis decreased IFNγ expression by Vγ9Vδ2 T cells in contact with A549 pretreated either with zoledronate or with BrHPP whilst blocking ICAM-1/LFA-1 or CD31/CD38 had no effect (Figure 2C). On the contrary, the weak Vγ9Vδ2 T cell activation by PC9 was decreased at the level of the ICAM-1/LFA-1 axis, whilst blocking of LFA-3/CD2 and of CD31/CD38 had no effect (Figure 2D). Moreover, neither the blocking of NKG2D nor PD1 had an impact on Vγ9Vδ2 T cell activation by A549 or PC9.

Finally, A549 and PC9 displayed different patterns of surface ligands and adhesion molecules and involved different surface molecules in PAg-dependent activation of Vγ9Vδ2 T cells. However, these differences cannot be sufficient to explain the different activation of Vγ9Vδ2 T cells by PC9 compared to A549. Indeed, PC9 expresses a high proportion of the inhibitory PDL1 and a high amount of ICAM-1 and ULBPs which are supposed to favor a productive immunological synapse. Conversely, A549 expresses CD38 but also expresses the inhibitory ligand HLA-A,B,C and a weak amount of ULBPs.

As the difference in surface markers of the two NSCLC cell lines did not explain the difference in Vγ9Vδ2 T cells activation, we decided to explore inside the cells.

### RHOB Deletion in PAg-Treated A549 Cell Lines Decreases Vγ9Vδ2 T Cell Activation

**Figure 3:**
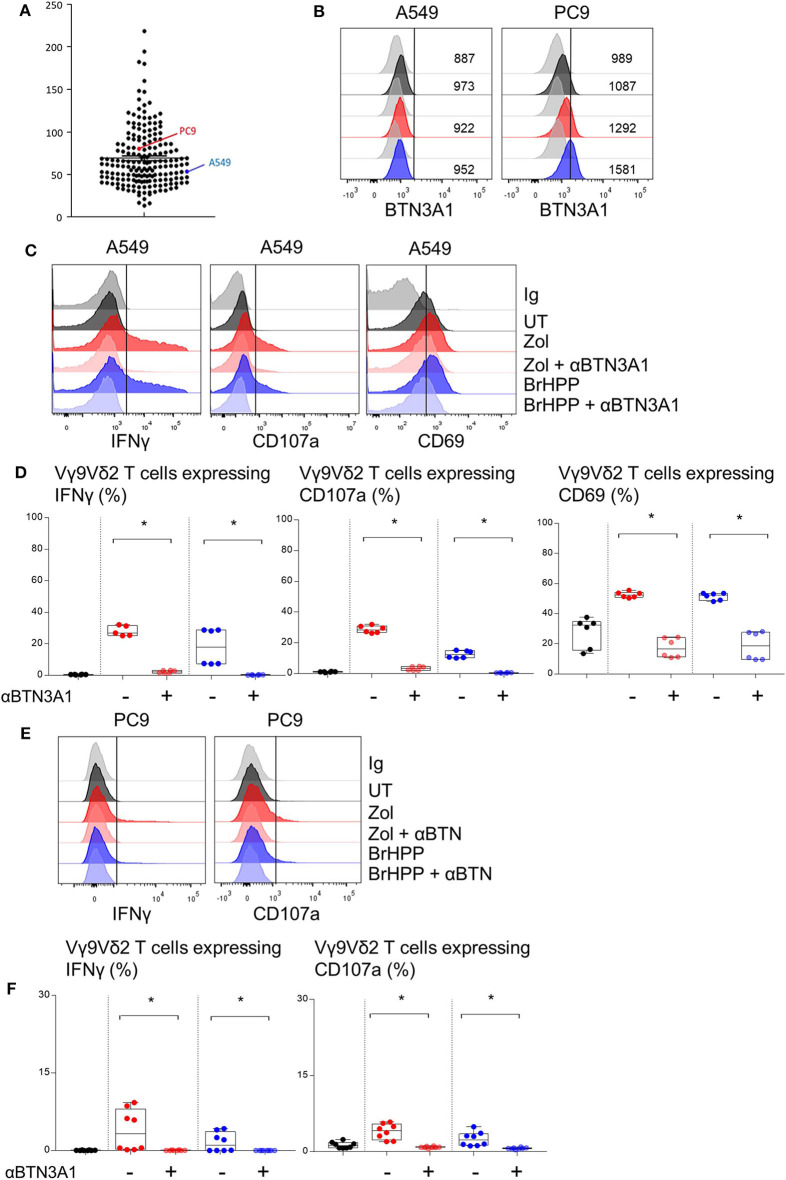
PAg-treated A549/PC9 cell lines activating Vγ9Vδ2 T cells is BTN3A1 dependent. **(A)** mRNA expression of the BTN3A1 by A549 and PC9 cell lines among cancer cell lines in the Cancer Cell Line Encyclopedia (CCLE). **(B)** Flow cytometry analysis of the BTN3A1 expression by A549 and PC9 pre-treated or not (black) by zoledronate (red) or BrHPP (blue) and compared to the isotypic control (gray). **(C–F)** Flow cytometry analysis of the IFNγ, CD107a, and CD69 expression by Vγ9Vδ2 T cells in co-culture for 4 h with A549 [**(C,D)**: six independent experiments] or PC9 [**(E,F)**: six independent experiments] without pretreatment (black) or with zoledronate (red) or BrHPP (blue) pretreatment. *indicates *p* < 0.05.

**Figure 4:**
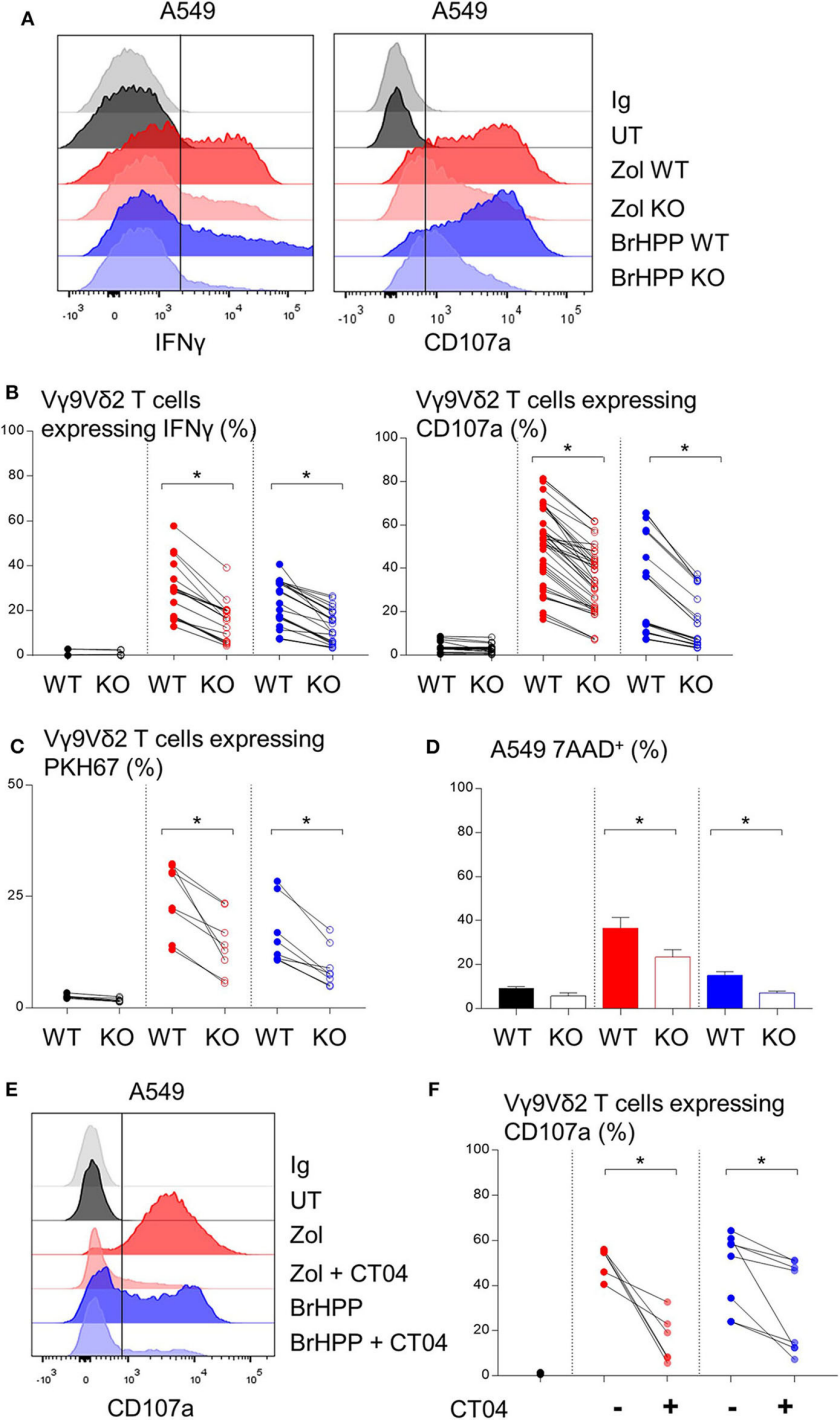
Decrease of the Vγ9Vδ2 T cells activation by A549 with RHOB knock down. Flow cytometry analysis of the IFNγ and CD107a expression by Vγ9Vδ2 T cells in co-culture for 4 h with A549 wild type (WT) or knock down for RHOB (KO) with zoledronate (red) or BrHPP (blue) pretreatment [**(A)**: one representative experiment, **(B)**: *n* > 10 independent experiments]. **(C)** Flow cytometry analysis of the trogocytosis of the PKH67^+^ PAg-pretreated-A549 cell line WT or KO by the Vγ9Vδ2 T cells (ratio of the Vγ9Vδ2 T cells expressing PKH67 4 h/5 min). **(D)** Flow cytometry analysis of the 7AAD expression by the WT or KO A549 cell line PAg-treated (zoledronate: red, BrHPP: blue) or not (black) in co-culture for 4 h with the Vγ9Vδ2 T cells. **(E,F)** CD107a expression by the Vγ9Vδ2 T cells analyzed by flow cytometry after 4 h of contact with PAg-pretreated A549 in the presence or not of the CT04 inhibitor [**(E)**: one representative experiment, **(F)**: *n* > 4 independent experiments]. *indicates *p* < 0.05.

BTN3A1 was previously shown to be involved in Vγ9Vδ2 T cell TCR-dependent activation. BTN3A1 may interact with the Vγ9Vδ2 TCR upon association with RHOB activated by its interaction with endogenous phosphoantigens (18). Thus, we investigated the role of RHOB in Vγ9Vδ2 T cell activation by these NSCLC cell lines. Firstly, A549 and PC9 cell lines were shown to express BTN3A1 at the transcriptomic and protein level (Figures 3A,B). Interestingly, PAg treatment of these cell lines had no impact on their BTN3A1 expression. We then determined whether Vγ9Vδ2 T cell activation by zoledronate- or BrHPP-treated NSCLC cell lines was BTN3A1-dependent using a blocking antibody (103.2 clone). The percentage of Vγ9Vδ2 T cells expressing IFNγ, CD107a and CD69 following contact with BrHPP- or zoledronate-treated A549 was highly decreased by the presence of anti-BTN3A1 during the co-culture (Figure 3C for a representative experiment and Figure 3D for pooled experiments). BTN3A1 blocking also abrogated the weak activation of Vγ9Vδ2 T cells by the PC9 cell line (Figure 3E for a representative experiment and Figure 3F for pooled experiments). Thus, the high activation of Vγ9Vδ2 T cells by PAg-treated A549 and the weak activation by PAg-treated PC9 are totally dependent on BTN3A1. As RHOB has been implicated in the regulation of the immune response through BTN3A1 modulation (18), we studied the implication of this GTPase in Vγ9Vδ2 T cell activation by NSCLC cell lines. RHOB was knocked out in the A549 and PC9 cell lines by the TALEN gene silencing method (Supplementary Figure 5). We then assessed the effect of this knockdown (KO) on Vγ9Vδ2 T cell activation by measuring IFNγ and CD107a expression, and trogocytosis after co-culture with the PAg-treated RHOB KO A549 or the PAg-treated RHOB KO PC9. IFNγ and CD107a expression by Vγ9Vδ2 T cells was lower in the co-culture with A549 RHOB KO treated either by zoledronate or BrHPP compared to the co-culture with PAg-treated A549 wild type (WT) (Figure 4A for a representative experiment and Figure 4B for pooled experiments). Trogocytosis of A549 by Vγ9Vδ2 T cells was also reduced when RHOB was knocked down which was correlated with reduced death of A549 RHOB KO compared to A549 WT (Figures 4C,D).

Furthermore, we used a RHO GTPase inhibitor, the exoenzyme C3 transferase (CT04) that specifically inhibits RHOA, B, C. We showed that inhibition of RHO GTPases abrogated CD107a expression by Vγ9Vδ2 T cells in co-culture with A549 previously treated with zoledronate or BrHPP (Figure 4E for a representative experiment and Figure 4F for pooled experiments).

Moreover, the weak activation of Vγ9Vδ2 T cells by PAg-treated PC9 was also dependent on RHOB (Supplementary Figure 6).

### Rerouting of RHOB in Endosomal Clusters Could Impair the PAg-Dependent Vγ9Vδ2 T Activation in PC9 Cells

**Figure 5:**
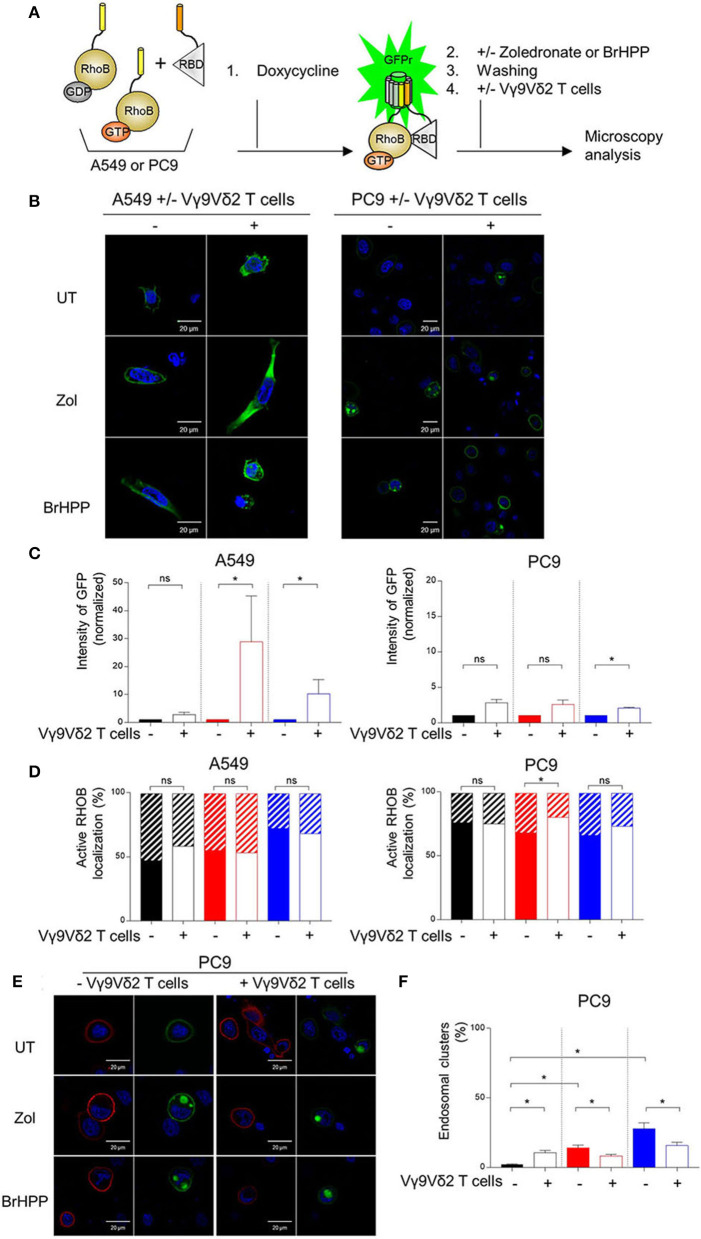
Membrane localization of RHOB favored in A549 cells in contact with Vγ9Vδ2 T cells. **(A)** A549 and PC9 cells were engineered to stably express RHOB GTPase and monitor active RHOB using a split GFP system. Induction by Doxycycline of the tripartite split-GFP system in A549 and PC9, with or without (UT) pretreatment with zoledronate (zol) or BrHPP, and co-cultured or not with Vγ9Vδ2 T cells. Active RHOB (green fluorescence) was visualized by confocal microscopy **(B)** and quantified by Operetta [**(C)**: zoledronate: red, BrHPP: blue]. **(D)** Percentage of endosomal (hatched bars) or membrane (full bars) active RHOB quantified by confocal microscopy in A549 and PC9 cell lines PAg-treated (zoledronate: red, BrHPP: blue) or not (black) co-cultured with Vγ9Vδ2 T cells. **(E,F)** Confocal microscopy of total (red) and active (green) RHOB in PC9 cell line after PAg-treatment (zoledronate: red, BrHPP: blue) or not (black) and co-cultured or not with Vγ9Vδ2 T cells [**(E)**: representative images; **(F)**: % of PC9 presenting endosomal clusters based on 3 independent experiments among 30 cells]. *indicates *p* < 0.05, Student's paired *t*-test; ns: no significant.

**Figure 6:**
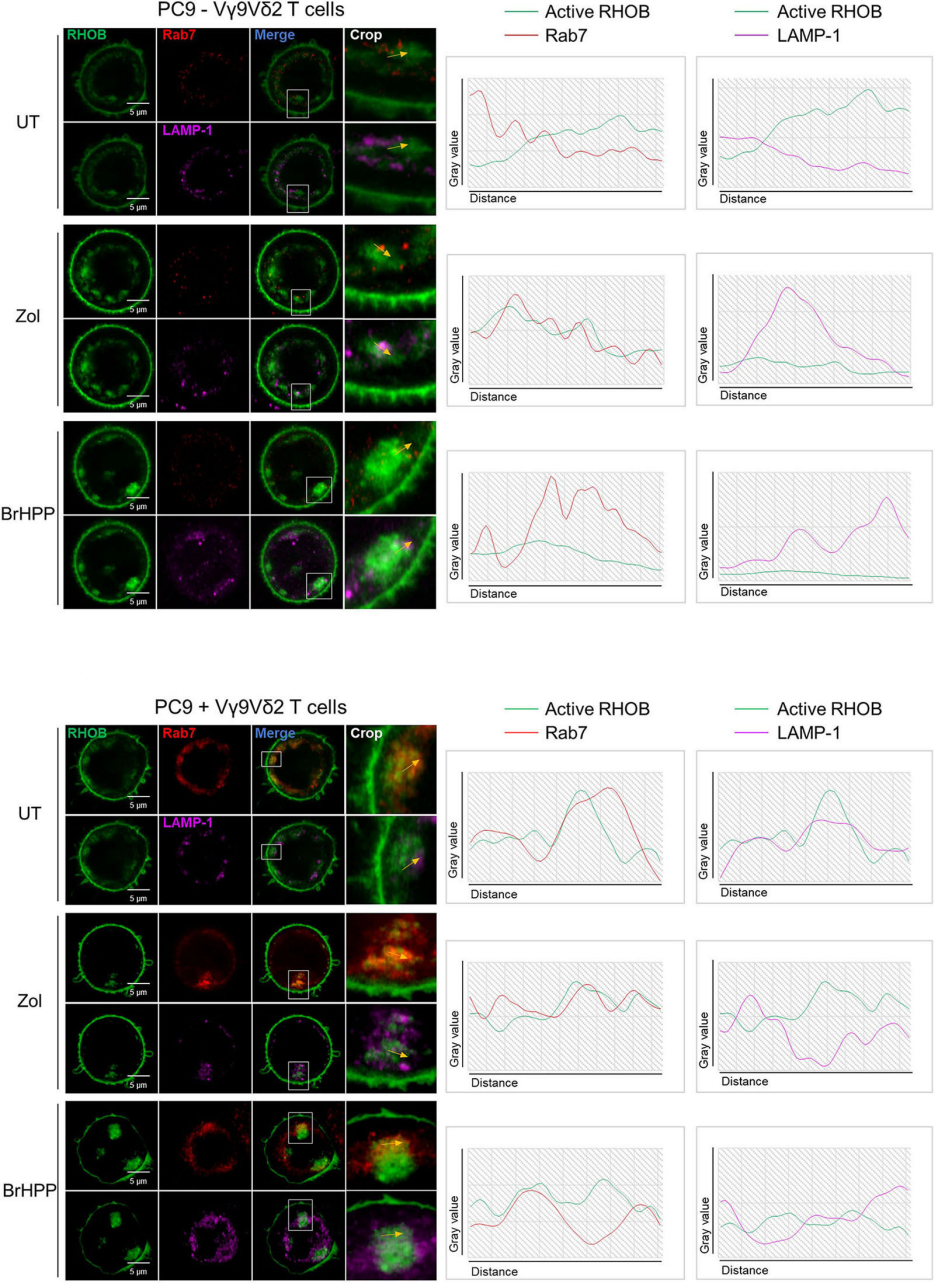
Rerouting of RHOB in endosomal clusters in PAg-treated PC9 cells in contact with Vγ9Vδ2 T cells. Co-localization analysis by confocal microscopy of active RHOB (green), Rab7 (red) for late endosome/MVB marker and LAMP-1 (pink) as lysosomal marker, in PC9 cells pre-treated or not (UT) with zoledronate (zol) or BrHPP, and co-cultured (lower) or not (upper) with Vγ9Vδ2 T cells (representative image, crop on the key zone, and plot profile analysis beside showed by yellow arrows).

We then postulated that the PAg-treatment of tumor cell lines and/or their co-culture with Vγ9Vδ2 T cells could have an impact on RHOB activity using a fluorescent reporter based on the tripartite split-GFP system (22) which monitors the binding of activated RHOB to RBD, one of its effector domains (20). A549 and PC9 cells were engineered to stably express RHOB GTPase fused to strand 10 of trisfGFP (GFP10-RhoB) and the Rho-binding domain of Rhotekin (RBD) fused to strand 11 (RBD-11). The GFP1-9 detector fragment allows detection of the RHOB-GTP and RBD interaction (Figure 5A). Reporter cell lines were pre-treated with zoledronate (5 μM, overnight) or BrHPP (1 μM, 4 h) and co-cultured or not with Vγ9Vδ2 T cells. Treatment of A549 with these PAg induced and increased the number of fluorescent cells and their fluorescence intensity only in the presence of Vγ9Vδ2 T cells indicating a significant increase of RHOB activity in this tumor cell line (Figure 5B, representative images and Figure 5C quantification). Conversely, in PC9 cells, RHOB activity was not modulated by PAg treatment and slightly modulated in co-culture with Vγ9Vδ2 T cells, suggesting a different RHOB regulation mechanism in this cell line (Figures 5B,C). To take a closer look at RHOB function, we performed confocal imaging in untreated and PAg-treated conditions, in the presence or not of Vγ9Vδ2 T cells and we analyzed the distribution of active RHOB (Figure 5D, membrane localization full bars and endosomal localization hatched bars). Surprisingly, active RHOB was significantly twice as abundant in the endosomal compartment of A549 cells compared to PC9 cells (Figure 5D, quantification). High resolution microscopy allowed us to analyze endosomal organization in PC9. Untreated reporter PC9 showed faint plasma membrane localization of RHOB/RBD complexes. Interestingly, treatment with zoledronate or BrHPP induced an accumulation of large endosomal clusters (Figure 5E, representative images). Quantification of the number of these structures indicated an increase of activated RHOB located in endosomal clusters in PAg-treated PC9 co-cultured or not with Vγ9Vδ2 T cells (Figure 5F). These endosomal clusters were reported randomly in PC9 cells co-cultured with Vγ9Vδ2 T cells independently of the treatment. Interestingly, these endosomal structures did not appear in the PAg-treated A549 co-cultured or not with Vγ9Vδ2 T cells (Supplementary Figure 7), suggesting that this endosomal reorganization was not due to the treatment with P-Ag mevalonate inhibitors. To identify the nature of these structures, we analyzed the localization of activated RHOB with different endosomal markers. Representative images and plot profile analysis on confocal stacks indicated a co-localization partly with late endosomal marker Rab7 and to a lesser extend with the LAMP1 lysosomal marker only after PAg-treated PC9 were co-cultured with Vγ9Vδ2 T cells independently of the treatment (Figure 6). These results indicate that in PC9 cells, endosomal RHOB is located in the late endosomal and on route to the lysosomal compartment.

## Discussion

The anti-tumor effect of Vγ9Vδ2 T cells depends on the phosphoantigens expressed by tumor cells but also on certain activator ligands (MICA/B and ULBPs) and adhesion molecules, essential to stabilize the immunological synapse. As PAg is not expressed by all tumor cells, treatment with exogenous PAg such as BrHPP or with aminobisphosphonates which induce endogenous PAg production such as isopentenyl pyrophosphate (IPP), is required to activate the anti-tumor functions of Vγ9Vδ2 T cells. However, Vγ9Vδ2 T cell activation can also be lowered by inhibitory signals that can be expressed by the tumor microenvironment and in particular by tumor cells. In this study, none of the NSCLC cell lines were able to spontaneously activate Vγ9Vδ2 T cells. However, pre-treatment with BrHPP or zoledronate sensitized some of them to Vγ9Vδ2 T cell killing. Indeed, amongst them, A549 was able to strongly activate IFNγ and CD107a expression by Vγ9Vδ2 T cells whereas PC9 induced a weak activation of these lymphocytes. Interestingly, these two cell lines express RHOB but wear different mutation, KRAS for A549 and EGFR for PC9. However, nothing was described concerning some phenotypic specificities which could explain the different sensitivity of these cells toward Vγ9Vδ2 T killers.

Surprisingly, exploring their surface molecule pattern, we showed that these two NSCLC cell lines could differentially express NKG2D ligands, immune checkpoint ligands, and adhesion molecules. PC9 highly expressed ICAM-1, ULBPs, and PDL1 whereas A549 highly expressed CD38 and HLA-A,B,C, the two cell lines expressed LFA-1, LFA-3, and CD155 but not ICAM-3, CD112, MICA/B, HLA-E, or galectin-9. The presence of ULBPs ligands could partially explain the reactivity of Vγ9Vδ2 T cells against NSCLC cell lines. However, blocking NKG2D expressed at the surface of Vγ9Vδ2 T did not decrease their activation which is thus only induced here by PAg and not by other activator ligands. Furthermore, we showed that blocking of ICAM-3/LFA-3 decreased the cytolytic activity of Vγ9Vδ2 T against A549 and blocking of ICAM-1/LFA-1 decreased this activity against PC9. For these two cell lines, stabilization of the immunological synapse around TCR/BTN3A1 is thus coordinated by two different adhesion systems. If LFA-1 engagement by ICAM-1 is sufficient to activate iNKT cells (23) in this model of lung cancer/Vγ9Vδ2 T, interactions of adhesion molecules are not sufficient to induce an activating signaling in Vγ9Vδ2 T cells. However, according to the literature, these interactions are necessary when their inhibition highly decreases Vγ9Vδ2 T activation (9, 24). But this does not explain the difference between A549 and PC9. First, we thought that the high expression of PDL1 by PC9 could explain the inhibition of Vγ9Vδ2 T activation. However, PD1 blocking did not induce an increase in CD107a or IFNγ expression by Vγ9Vδ2 T cells. As surface inhibitor markers were not at the origin of the weak activation of Vγ9Vδ2 T cells, we focused on RHOB inside the cells. According to the literature (18) and thanks to NSCLC cell lines with a RHOB KO, we showed that RHOB was involved in the Vγ9Vδ2 T activation through endogenous PAg induced by a zoledronate treatment but also through exogenous PAg such as BrHPP. The non-reactivity of Vγ9Vδ2 T cells with the conditioned medium of BrHPP pre-treated tumor cells or in co-culture with these cells separated by a porous membrane (Transwell), demonstrated that BrHPP was able to penetrate inside the tumor cell and to activate membrane BTN3A1 shown to be essential for this activation (19). RHOB is thus also involved in the activation of Vγ9Vδ2 T cells through the exogenous PAg, BrHPP. Interestingly, the ICAM-1 signaling cascade is also highly dependent on RHO proteins, as ICAM-1 crosslinking induces actin reorganization which involves RHOB proteins (25). PC9 expresses a high amount of ICAM-1 which is involved in the immunological synapse with Vγ9Vδ2 T but does not efficiently activate the latter. To explain the weak reactivity of Vγ9Vδ2 T cells against PAg-pretreated PC9, we examined RHOB activity and its localization. The similar reactivity of Vγ9Vδ2 T cells against A549 and PC9 pulsed with the 20.1 BTN3A1 agonist (Supplementary Figure 8) supports the important role of RHOB in the PAg-induced response in this model. Our results show that active RHOB is localized in endosomes and at the plasma membrane of both A549 and PC9 cell lines. This is in agreement with previous reports of GFP-RHOB fusions in human epithelial cells (26). Our results indicate that active RHOB is increased in PAg-treated A549 cells co-cultured with Vγ9Vδ2 T cells, whereas only a slight increase of active RHOB is observed in PC9 cells co-cultured with Vγ9Vδ2 T cells independently of PAg treatment. It is important to note that the activation of RhoB in co-culture of A549 and Vγ9Vδ2 T is mainly dependent on the treatment by the PAg (Figure 5C). It has been previously established that treatment with PAg induces an increase of active RHOB that will modify the conformation of the BTN3A1 at the plasma membrane, which allows the activation of Vγ9Vδ2 T cells (18). The fact that the presence of Vγ9Vδ2 T cells further increases RHOB activity suggests that RHOB may be activated by exogenous stress signals and cytokines that would come from activated Vγ9Vδ2 T cells. Indeed, in other contexts involving immune cells, RHOB has been shown to be upregulated by environmental stress, cytokines, and LPS, and regulating this latter signaling (27–29). Our hypothesis would be that signals secreted by activated Vγ9Vδ2 T cells would contribute to RHOB activation in a cooperative manner with PAg. These results corroborate with the Vγ9Vδ2 T cell activation observed with A549 or -PC9 treated with PAg (Figure 1 and Supplementary Figure 1). Altogether this indicates that the reactivity of these cell lines is strongly linked to RHOB function. In terms of subcellular localization, both cell lines contain active RHOB at the plasma membrane, with a stronger distribution in PC9 cells. A higher proportion of active RHOB is present in the endosomal compartment in A549 compared to PC9, however they differ strongly in terms of endosomal distribution. Whereas in A549, endosomal RHOB is diffusely distributed, in PC9 endosomal active RHOB was found in large clusters that co-localized with late Rab7 endosomal and LAMP-1 lysosomal markers. It is known that Vγ9Vδ2 T cell activation occurs at the plasma membrane through the involvement of BTN3A1 that is addressed by RHOB vesicles from the endosomes to the plasma membrane (18). In our model, PAg treatment of PC9 cells is not sufficient to trigger an increase in RHOB activation but requires the contact with Vγ9Vδ2 T cells, as untreated conditions in the presence of Vγ9Vδ2 T cells did not induce a significant increase in RHOB activity. One hypothesis of the weaker activation of RHOB in PC9 cells is that beyond the mode of recognition with T cells, the organization of endosomal RHOB-mediated signaling is not efficient for addressing important signaling molecules such as BTN3A1 at the plasma membrane. It was reported that RHOB interacts with the intracellular domain of BTN3A1 at the plasma membrane (18). BTN3A1 was shown as essential to Vγ9Vδ2 T cell recognition but not sufficient for this process as its homologous BTN2A1, the phosphoantigens being also essential. Actually, it was recently described that BTN2A1 synergized with BTN3A1 in sensitizing PAg-exposed cells for Vγ9Vδ2 TCR-mediated responses, these two butyrophilins beings key ligands that bind possibly two different domains of this TCR (30, 31). As for BTN3A1, the level of expression of BTN2A1 mRNA in PC9 is very close to that in A549 (Supplementary Figure 9). Thus, we can expect that these two cell lines express a similar level of BTN2A1 at their membrane, the BNT3A1 expression being equivalent (Figure 3B). Therefore, the weak activation of the Vγ9Vδ2 T cell by the PC9 cell line should not be due to the lack of BTN2A1. RHOB has not yet been shown to be associated to the intracellular B30.2 domain of the BTN2A1. Actually, it could be interesting to know if RHOB, as for BNT3A1, can be involved in the modification of the conformation of the BTN2A1. The organization of endosomal RHOB could then have an impact also on the BTN2A1. Further investigations should be pursued to evaluate whether such a mechanism is preserved in PC9. This endosomal signaling could be favored due to the genetic background of these cells, i.e., as they express an activated EGFR mutant whose expression may modify intracellular RHOB mediated trafficking. Indeed, RHOB is known to prolong endosomal signaling of EGFR following its internalization (32). RHOB is a short-lived protein that is rapidly degraded through the endo-lysosomal pathway (33) and its degradation is delayed by inhibition of its isoprenylation. Treatment with PAg, while necessary for the activation of Vγ9Vδ2 T cells, contributes to a rerouting of RHOB in late/degradation compartments that may accelerate its degradation and prevent its function in endocytic trafficking to the plasma membrane in PC9 cells.

This study demonstrates for the first time that Vγ9Vδ2 T cell activation by PAg-treated tumor cells can be variable depending on several factors such as oncogenic mutation, RHOB activity, and surface markers. Our results indicate that this response is strongly influenced by RHOB function. Therefore, the regulation of endocytic traffic by RHOB in the tumor cell could be decisive for the immune response and may explain the resistance of some tumor cells that nevertheless highly express RHOB.

## Data Availability Statement

The datasets presented in this study can be found in online repositories. The names of the repository/repositories and accession number(s) can be found below: https://portals.broadinstitute.org/ccle/data, CCLE.

## Author's Note

The precise mechanism of Vγ9 T cells phosphoantigen (PAg)-activation remains elusive even the butyrophilin BTN3A and the RHOB GTPase are known as essential in this activation. RHOB which can have a dualistic role in cancer, was shown as conferring resistance to EGFR-tyrosine kinase inhibitors in lung cancer and frequently downregulated in aggressive lung cancer. Besides, Vγ9 T cells based therapies could be an issue for advanced lung cancers refractory to or intolerant of current conventional treatment. The role of RHOB in the PAg-activation of Vγ9 T cells in lung cancer has to be depicted when Vγ9 T cells reactivity depends on the lung tumor cell lines status.

## Author Contributions

CLap, SM, MM, and SF performed the experiments. SF and OC performed the KO cell lines. SC, OC, J-JF, GF, and CLau participated to the discussion of the results. MP and SC designed experiments and wrote the manuscript. MP supervised the study. All authors contributed to the article and approved the submitted version.

## Conflict of Interest

The authors declare that the research was conducted in the absence of any commercial or financial relationships that could be construed as a potential conflict of interest.
