# Supplementary material for: Vγ9Vδ2 T Cells Activation Through Phosphoantigens Can Be Impaired by a RHOB Rerouting in Lung Cancer
Source: Front Immunol. 2020 Jul 7;11:1396. doi: 10.3389/fimmu.2020.01396 (PMC7358576; doi:10.3389/fimmu.2020.01396)
Supplement: Supplementary file 1 [file Data_Sheet_1.PDF]

Supplementary figure 1

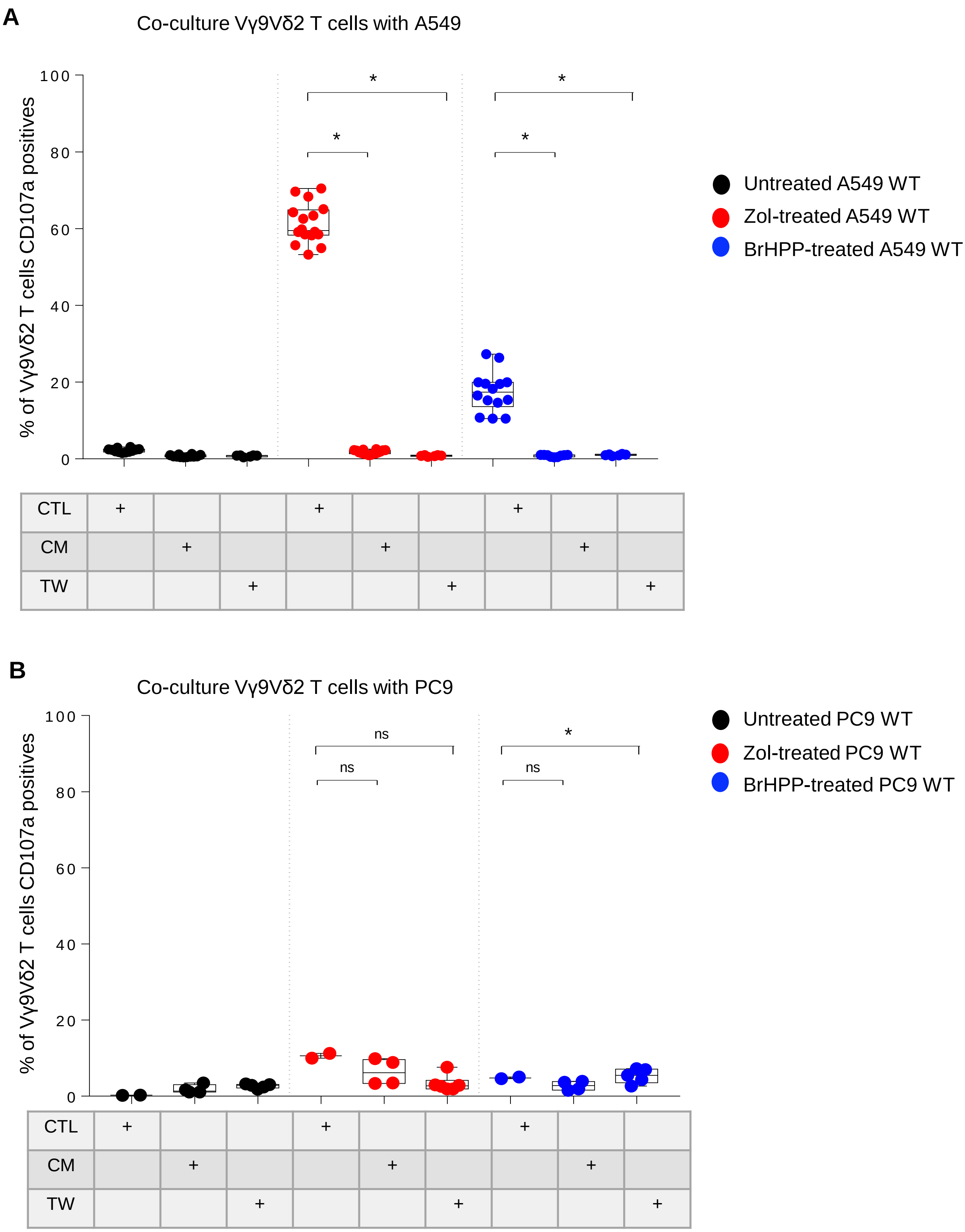

Percentage of Vγ9Vδ2 T cells expressing CD107a, analyzed by flow cytometry after 4h of contact with PAg-pretreated A549 (**A**) or PC9 (**B**) in the presence of a Transwell system (TW) or not (CTL) or in the presence of the conditioned medium of these PAg-treated cells lines (CM) (n > 4 independent experiments).

Supplementary figure 2

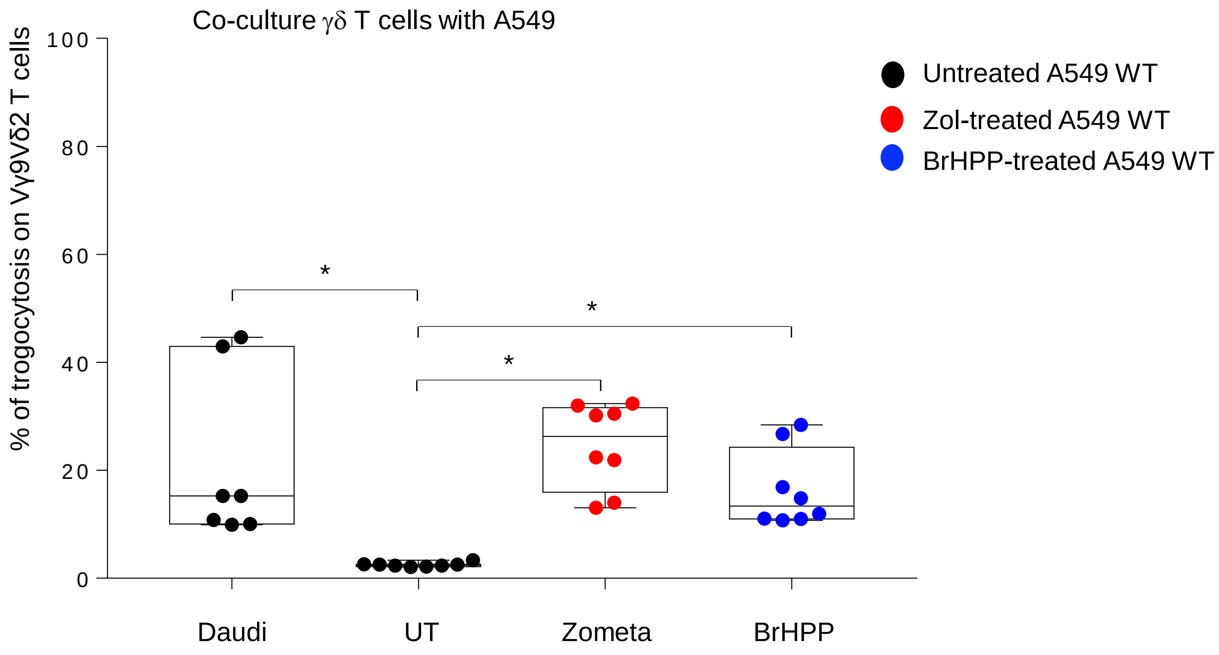

Percentage of Vγ9Vδ2 T cells expressing PKH67 (trogocytosis), analyzed by flow cytometry, after a co-culture with beforehand PKH67 stained Daudi or A549 pretreated or not (UT) by zoledronate (red dots) or BrHPP (blue dots) (n > 4 independent experiments).

Supplementary figure 3

A

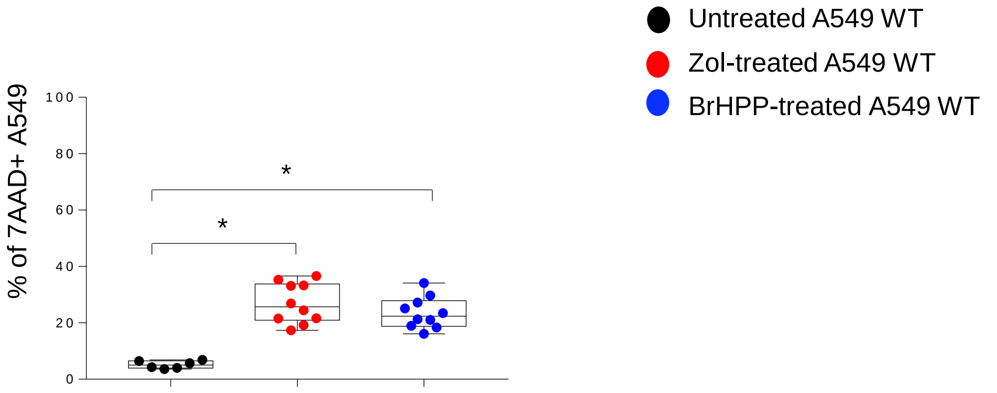

B

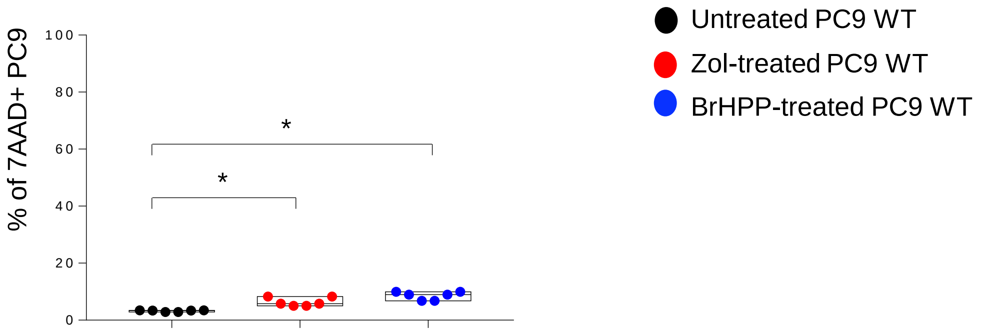

Percentage of A549 (A) or PC9 (B) pretreated or not (black dots) by zoledronate (red dots) or BrHPP (blue dots) expressing 7-AAD, after a co-culture with Vγ9Vδ2 T cells (flow cytometry analysis). (n > 4 independent experiments).

Supplementary figure 4

A

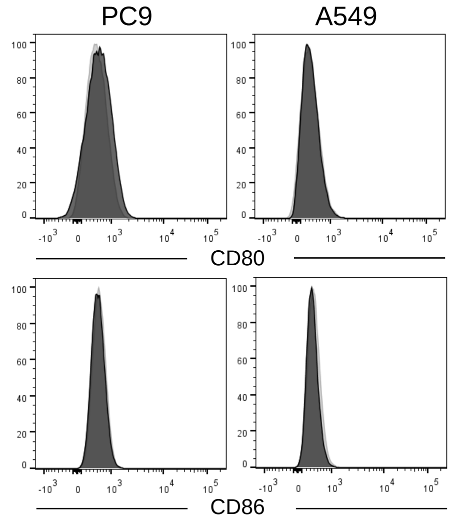

Surface molecule expression by A549 and PC9 untreated (black) or after treatment with zoledronate (red) or BrHPP (blue) analyzed by flow cytometry.

Untreated
Zometa-treated
BrHPP-treated

B

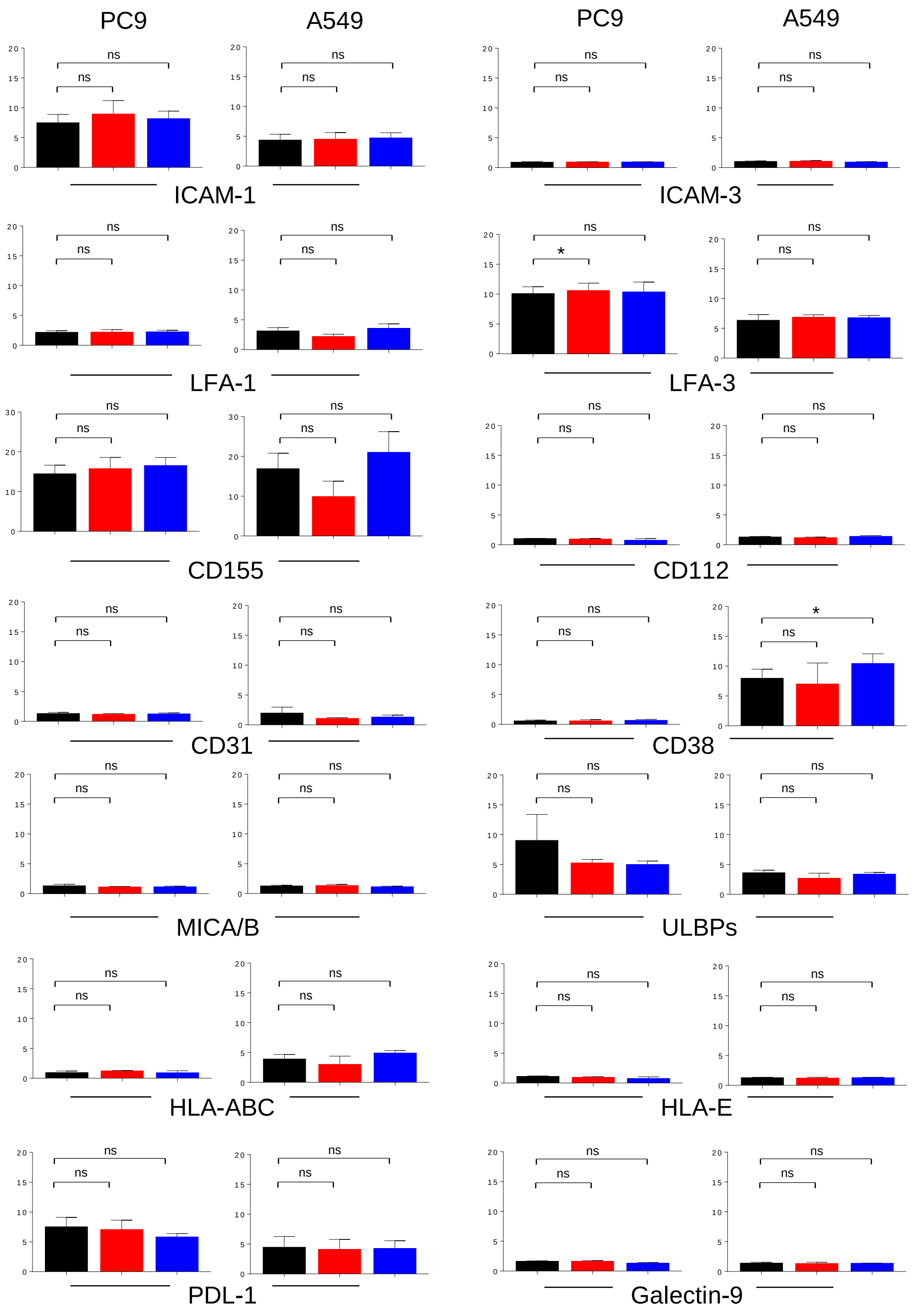

Supplementary figure 5

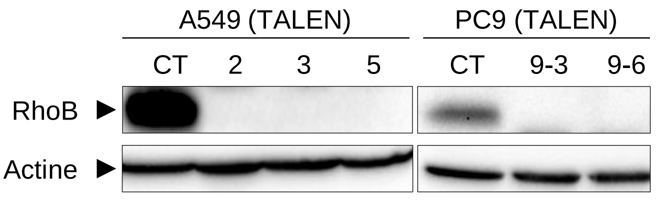

Western blot of A549 and PC9 wild type (CT) or KO for RHOB (2, 3, 5, 9-3, 9-6).

Supplementary figure 6

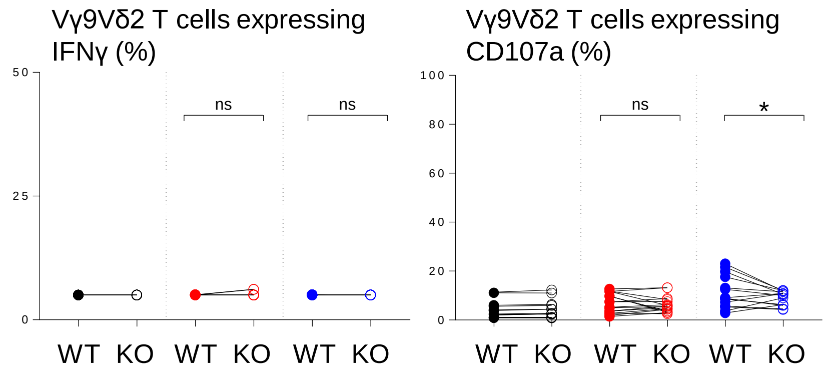

Flow cytometry analysis of the IFNγ and CD107a expression by Vγ9Vδ2 T cells in co-culture for 4h with PC9 wild type (WT) or knock down for RHOB (KO) with Zoledronate (red) or BrHPP (blue) pretreatment.

Supplementary figure 7

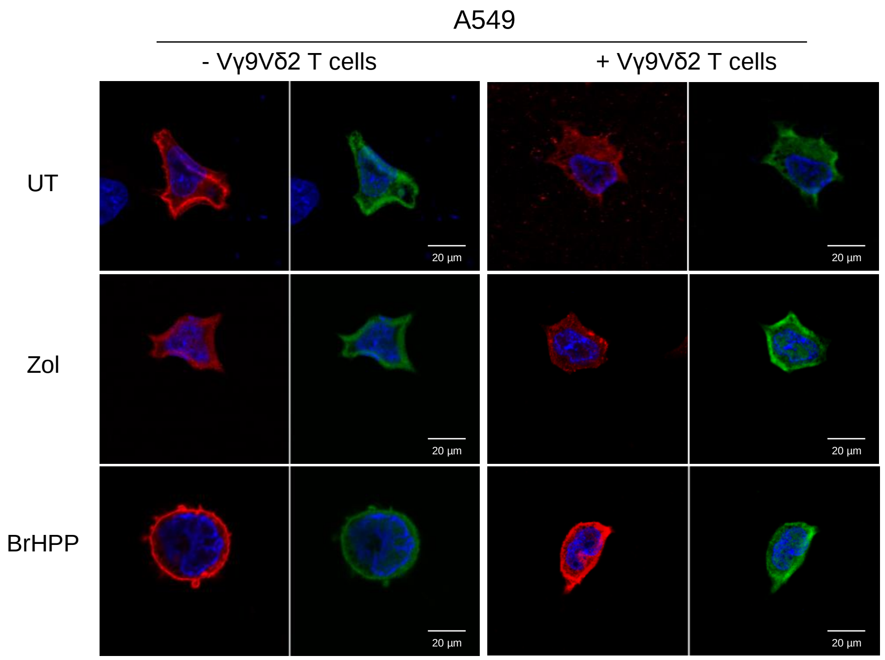

Confocal imaging of total (red) and active (green) RHOB localization in A549 pre-treated or not by zoledronate (5µM, overnight) or BrHPP (1µM, 4h), in presence or not of Vγ9Vδ2 T cells (one representative image).

Supplementary figure 8

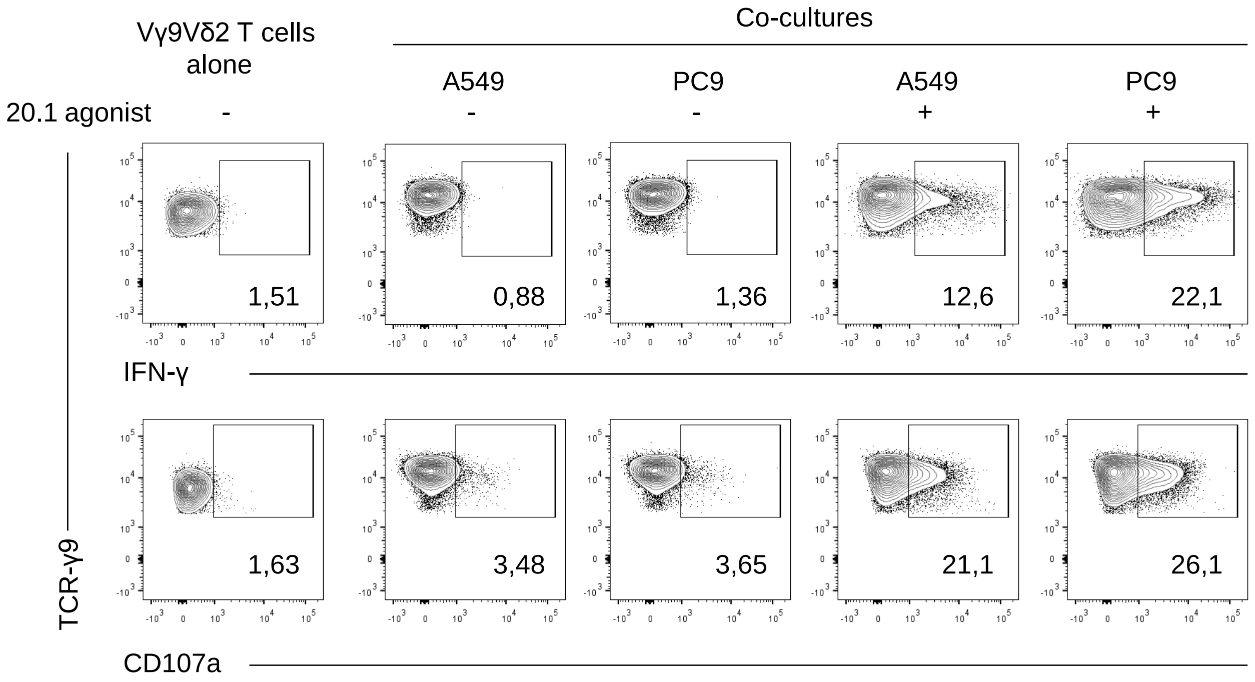

Percentage of Vγ9Vδ2 T cells expressing IFN-γ and CD107a, analyzed by flow cytometry, after co-culture for 4h with A549 or PC9 pretreated or not by the 20.1 BTN3A1 agonist.

Supplementary figure 9

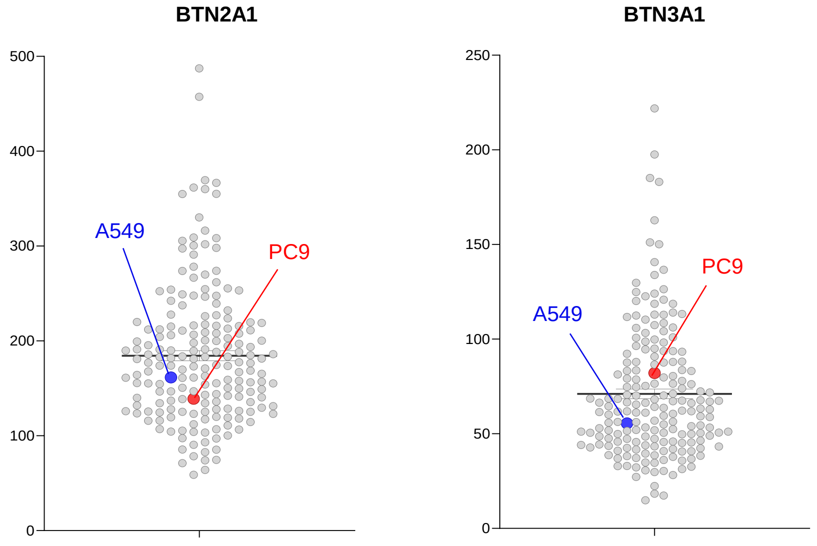

mRNA expression of BTN2A1 compared to BTN3A1 in A549 (blue) and PC9 (red) from Cancer Cell Line Encyclopedia.
